# MHC diversity and female age underpin reproductive success in an Australian icon; the Tasmanian Devil

**DOI:** 10.1038/s41598-018-20934-9

**Published:** 2018-03-08

**Authors:** Tracey Russell, Simeon Lisovski, Mats Olsson, Gregory Brown, Rebecca Spindler, Amanda Lane, Tamara Keeley, Chris Hibbard, Carolyn J. Hogg, Frédéric Thomas, Katherine Belov, Beata Ujvari, Thomas Madsen

**Affiliations:** 1 School of Life and Environmental Sciences, University of Sydney, Sydney, 2006 NSW Australia; 2 University of California Davis, Department of Neurobiology Physiology and Behavior, Davis, CA 95616 USA; 3 Dept of Biological and Environmental Sciences, University of Gothenburg, Box 463, SE 405 30, Gothenburg, Sweden; 4 Taronga Conservation Society Australia, Mosman, 2088 NSW Australia; 5 School of Agriculture and Food Science, University of Queensland, Gatton, 4343 QLD Australia; 6 Zoo and Aquarium Association Australasia, Mosman, 2088 NSW Australia; 7 CREEC/MIVEGEC, UMR IRD/CNRS/UM 5290, 911 Avenue Agropolis, BP 64501, 34394 Montpellier Cedex 5, France; 8 Centre for Integrative Ecology, School of Life and Environmental Science, Deakin University, Geelong, 3216 VIC Australia; 9 School of Biological Sciences, University of Wollongong, Wollongong, 2522 NSW Australia

## Abstract

Devil Facial Tumour Disease (DFTD), a highly contagious cancer, has decimated Tasmanian devil (*Sarcophilus harrisii*) numbers in the wild. To ensure its long-term survival, a captive breeding program was implemented but has not been as successful as envisaged at its launch in 2005. We therefore investigated the reproductive success of 65 captive devil pair combinations, of which 35 produced offspring (successful pairs) whereas the remaining 30 pairs, despite being observed mating, produced no offspring (unsuccessful pairs). The devils were screened at six MHC Class I-linked microsatellite loci. Our analyses revealed that younger females had a higher probability of being successful than older females. In the successful pairs we also observed a higher difference in total number of heterozygous loci, i.e. when one devil had a high total number of heterozygous loci, its partner had low numbers. Our results therefore suggest that devil reproductive success is subject to disruptive MHC selection, which to our knowledge has never been recorded in any vertebrate. In order to enhance the success of the captive breeding program the results from the present study show the importance of using young (2-year old) females as well as subjecting the devils to MHC genotyping.

## Introduction

The major histocompatibility complex (MHC) has been shown to be one of the key molecular determinants of mate choice in numerous vertebrates including humans^1–7^. Class I and II MHC molecules are responsible for the processing and presentation of intra- and extra-cellular peptides derived from invading pathogens to cytotoxic T cells and helper T cells and, hence, constitute a crucial part of the vertebrate immune system^8,9^. Due to the ability to recognize and present peptides from a wide array of rapidly evolving pathogens, the MHC encompasses the most variable set of genes with heterozygosity values exceeding those predicted by neutrality^10^.

Pathogen-driven selection has been documented to result in two, not mutually exclusive, MHC responses: (i) selection for specific MHC alleles^11–14^ and/or (ii) selection for enhanced MHC polymorphism^13–16^. Although increased levels of MHC polymorphism enable wider recognition of pathogens, it might also lead to an inability to eliminate T cells reacting with self-peptide-MHC combinations^17,18^. Consequently, diversifying MHC selection might be counteracted by the deleterious effects caused by autoimmunity^19^. Indeed, theoretical models as well as empirical studies have shown that optimal pathogen resistance often occurs at an optimal intermediate level of MHC polymorphism^13,20–27^.

MHC has also been shown to be involved in individual mate choice and providing offspring with indirect genetic benefits in at least three ways via (i) acquisition of “good genes” i.e. genetic elements that contribute to lifetime reproductive success regardless of an individual’s additional genotype^28^, and/or (ii) acquiring optimal genetic compatibility^29^, and/or (iii) achieving enhanced genetic diversity^30^.

Importantly, significant reduction in MHC diversity may not only affect resistance to pathogens but has also been shown to result in an increased risk of extinction due to inbreeding depression^31^. Moreover, reduced MHC polymorphism has been suggested to have contributed to the emergence of a clonally transmissible cancer, Devil Facial Tumour Disease (DFTD) in the world’s largest living marsupial carnivore: the Tasmanian devil (*Sarcophilus harrisii*)^32^. DFTD was first reported in north-eastern Tasmania in 1996 but since then has caused severe reductions in devil numbers (>70%) questioning the long-term survival of this iconic species^33,34^. This devastating disease is spread among devils via biting during social interactions^35^. The devil’s immune system is unable to mount an effective immune response to the tumour as DFTD cells are able to avoid immune recognition by down-regulating MHC expression^36,37^. Metabolic failure, tumour related cachexia and metastases result in devil death within 6 to 9 months of the emergence of the first lesions^38^. In order to ensure that the Tasmanian devil will not face extinction a large scale captive breeding program commenced in 2005^39^, but overall breeding success is still not optimal^40,41^. In the present study we therefore explore how traits such as devil age and MHC polymorphism affected reproductive success in the captive devil population.

## Results

**Figure 1:**
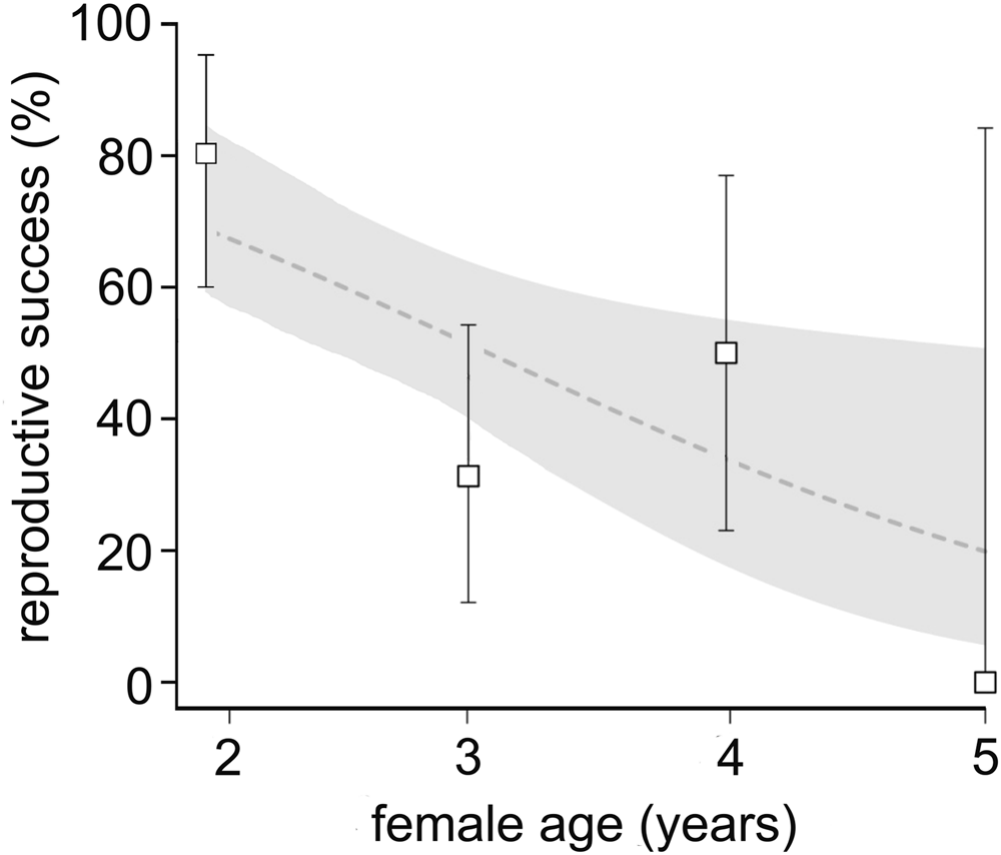
Relationship between female age and the success of reproduction in Tasmanian devil pairs (**a**). Points with error bars depict reproductive success (±95% CI) of pairs across female age. Grey line shows the binomial model prediction of this relationship with the 95% confidence interval (grey polygon).

**Figure 2:**
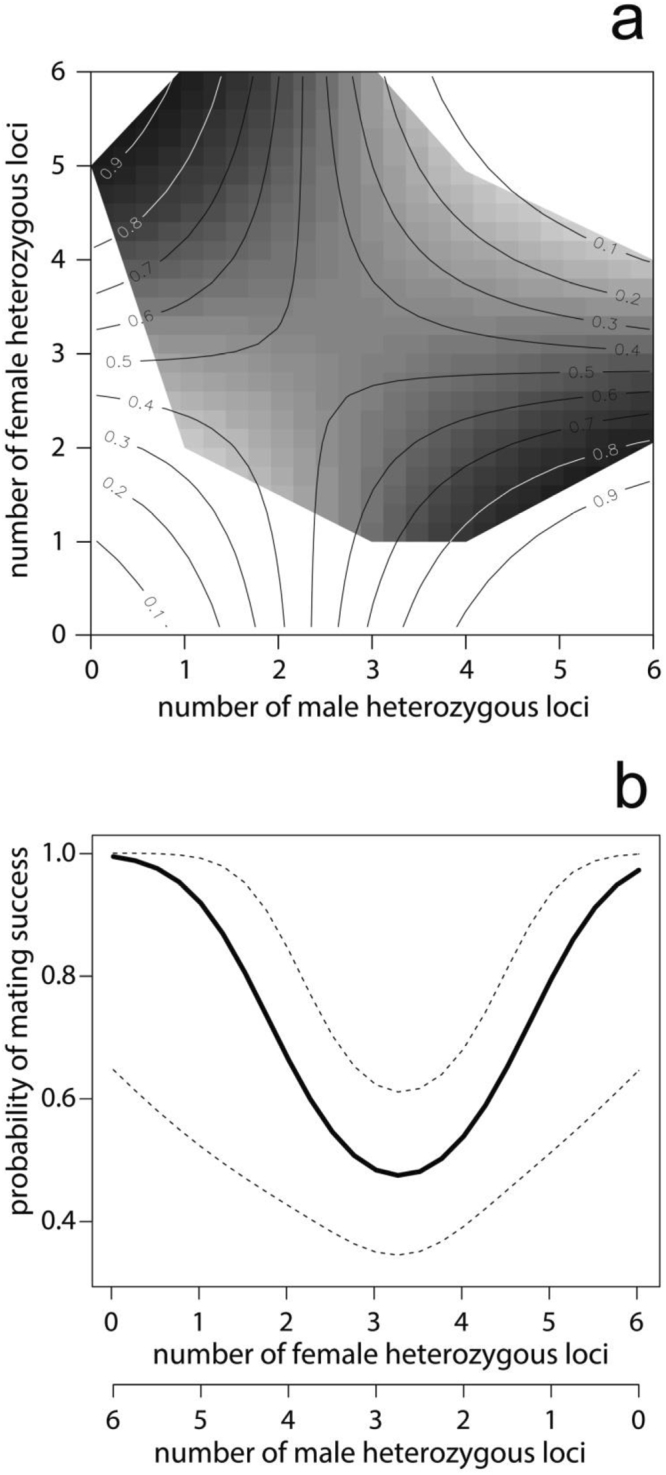
(**a**) Mode prediction of the interaction between the number of male and female heterozygous loci. The color scale (within the data range) and contour lines (across the entire range of female and male heterozygous loci) indicate the probability of being successful according to the combination of the number of male and female heterozygous loci. (**b**) Diagonal section of the model prediction shown in (**a**) across the optimal pair combinations with 95% confidence interval.

Of a total of 65 captive devil pair combinations, 35 produced offspring whereas the remaining 30 pair combinations did not produce any offspring (see Table 1 for a detailed description of the pair combinations). The genetic diversity analyses did not reveal any allele dropouts or null alleles. The full logistic mixed model revealed that male age, identification number, number of female heterozygous loci, number of male heterozygous loci and number of similar alleles did not affect devil reproductive success (P > 0.20), and those predictors were therefore backwards-eliminated. The final model revealed that younger females had a higher probability of being successful than older females as indicated by a negative regression parameter estimate for female age = −0.1068 ± 0.04, SE, p = 0.012 (Table 2). In fact, the final analysis predicted a decrease in the chance of producing offspring from 0.68 (0.82–0.51, 95% confidence interval) for females at the age of 24 months to 0.19 (0.5–0.05) for females older than 60 months (Fig. 1). As mentioned above, male age did not affect reproductive success. Interestingly, in successful pairs we observed a higher difference in total number of heterozygous loci, i.e. when one devil had a high total number of heterozygous loci, its partner had a low number, as evident by a positive regression parameter estimate (0.8202 ± 0.3329, p = 0.0165). Thus, pairs with opposing total numbers of heterozygous loci were found to have a higher probability of being successful reproducers than pairs with similar numbers (Fig. 2).

**Table 1:** Female – male pair combinations.

number of female heterozygous loci	female age (months)	female ID	number of male heterozygous loci	male age (months)	male ID	pair combination
4	48	FD1	1	48	MD1	successful
4	36	FD1	2	36	MD6	unsuccessful
4	36	FD1	3	36	MD21	unsuccessful
3	24	FD10	2	36	MD40	unsuccessful
4	36	FD11	1	60	MD8	successful
4	24	FD11	3	36	MD22	successful
6	48	FD12	1	60	MD8	successful
6	24	FD12	3	36	MD10	successful
3	24	FD13	4	24	MD15	unsuccessful
3	24	FD13	3	36	MD27	unsuccessful
4	36	FD14	2	24	MD30	unsuccessful
4	36	FD14	2	36	MD31	unsuccessful
4	36	FD14	3	24	MD37	unsuccessful
4	36	FD14	3	48	MD41	unsuccessful
4	24	FD15	1	24	MD32	successful
6	24	FD16	2	24	MD11	successful
6	48	FD16	3	48	MD43	successful
5	24	FD17	1	36	MD18	successful
5	36	FD17	2	36	MD31	successful
3	36	FD18	1	24	MD8	unsuccessful
3	24	FD18	4	48	MD15	successful
1	24	FD19	4	36	MD5	successful
1	36	FD19	3	36	MD33	successful
5	24	FD2	2	36	MD29	successful
5	24	FD20	1	36	MD8	successful
5	48	FD20	1	36	MD18	unsuccessful
5	48	FD20	2	36	MD19	unsuccessful
2	24	FD21	3	24	MD34	successful
4	48	FD22	3	48	MD43	successful
5	24	FD23	0	24	MD36	successful
3	24	FD24	4	24	MD12	successful
1	36	FD25	1	60	MD1	unsuccessful
1	24	FD25	4	24	MD38	successful
1	36	FD25	3	36	MD39	unsuccessful
2	24	FD26	4	24	MD12	successful
5	36	FD27	4	48	MD3	unsuccessful
5	24	FD27	1	24	MD32	unsuccessful
1	24	FD28	4	36	MD17	successful
2	36	FD29	4	48	MD26	unsuccessful
2	48	FD29	2	48	MD31	unsuccessful
3	24	FD3	4	36	MD5	successful
5	36	FD30	2	24	MD40	successful
5	60	FD31	2	60	MD23	unsuccessful
3	36	FD32	6	48	MD16	unsuccessful
3	36	FD32	4	48	MD26	unsuccessful
3	36	FD33	2	36	MD4	successful
3	48	FD33	4	48	MD25	successful
3	24	FD34	4	36	MD3	successful
3	36	FD34	1	36	MD32	unsuccessful
1	24	FD35	4	24	MD12	successful
2	24	FD36	4	36	MD7	successful
3	24	FD37	1	24	MD8	unsuccessful
1	36	FD4	4	36	MD42	successful
4	48	FD5	3	36	MD13	successful
4	36	FD5	3	24	MD33	successful
3	48	FD6	4	48	MD2	unsuccessful
3	48	FD6	5	48	MD14	unsuccessful
3	48	FD6	5	48	MD35	unsuccessful
2	36	FD7	2	48	MD9	unsuccessful
2	24	FD7	2	36	MD20	successful
2	36	FD7	2	36	MD24	unsuccessful
5	24	FD8	4	36	MD38	successful
5	36	FD8	3	48	MD39	successful
3	60	FD9	2	60	MD23	unsuccessful
3	36	FD9	2	36	MD28	unsuccessful

**Table 2:** Final results of logistic mixed model.

Effect	Estimate	SE	DF	t	Pr > |t|
**Parameter estimate**					
Intercept	1.9734	1.1183	62	1.76	0.0826
Difference in number of heterozygous loci	0.6204	0.2539	62	2.44	0.0174
Female age	−0.0871	0.0316	62	−2.75	0.0077
**Type III Tests of Fixed Effects**					
Effect	Num DF	Den DF	F	Pr > F	
Difference in number of heterozygous loci	1	62	5.97	0.017	
Female age	1	62	7.58	0.008

## Discussion

Prior to the emergence of DFTD female devils in the wild commenced reproduction at an age of two years and thereafter produced offspring for the next three years, becoming senescent at an age of five to six years^33^. The results from the present study, however, show that a decline in captive female reproductive success may already occur at an age of three years suggesting that some captive females might be affected by reproductive senescence at an earlier age than that recorded in the wild.

Previous studies have shown that devils are subjected to low genetic diversity at both neutral (microsatellite) and coding genomic regions at the MHC^42–45^. The low level of genetic polymorphism has been suggested to be caused by population bottlenecks during the Pleistocene and Holocene^46,47^ and by pathogens such as a canine-distemper-like disease in the early twentieth century^44^. The genetic diversity of the captive devils used in the present study was similar to that recorded in devils captured in the wild^48^. As we did not observe any overall significant difference in genetic diversity among the successful and the unsuccessful pair combinations we find it unlikely that the difference in reproductive success was caused by a concomitant discrepancy in genetic diversity.

In vertebrates such as sand lizards (*Lacerta agilis*)^49^, savannah sparrows (*Passerculus sandwichensis*)^50^, southern dunlins (*Calidris alpina schinzii*)^51^, great tits (*Parus major*)^52^ and the cynomolgus macaque *(Macaca fascicularis*)^53^ reproductive success has been negatively linked to male - female genetic similarity. However, the results from the present study suggest that within-pair genetic similarity did not affect devil reproductive success.

As mentioned above, both theoretical and empirical studies have shown that selection for an intermediate and optimal number of MHC alleles may result in both increased reproductive success and immune function^13,20–27^. However, our results show that pairs with higher difference in total number of heterozygous loci had an increased probability of being successful than pairs with similar numbers of heterozygous loci.

If devils have been under selection for an intermediate, optimal number of MHC alleles (e.g., 6 alleles), we would expect reproductive success to be the same in pairs in which partners both have 3 heterozygous loci as in pairs in which one partner has 1 and the other 5 heterozygous loci. Our results therefore suggest that devil MHC may be subject to disruptive selection. Although similar MHC processes have been suggested to drive MHC evolution in allopatric taxa in different habitats^54^, it has to our knowledge, never been recorded to affect reproductive success.

We propose two, not mutually exclusive processes, to underpin the significant effect of selection for MHC-driven devil reproductive success: pre-copulatory and/or post-copulatory (cryptic) female choice of male sperm. As mentioned above, MHC-based pre-copulatory mate choice, sometimes based on olfactory clues, has been recorded in wide range of vertebrates including humans^1–7,55^. MHC-based post-copulatory female cryptic choice of male sperm has also been documented in several vertebrates such as fish^56^, lizards^49^, birds^57^ and mammals^58^. As female devils in both the successful and unsuccessful pair combinations were observed mating^59–65^, we suggest that the significant difference in reproductive success between the two groups might be caused by post-copulatory cryptic female choice. In externally fertilizing fishes, the ovarian fluid released by the female may bias fertilization success towards males with a particular genotype^66,67^. However, if a similar mechanism may influence male fertilization success in internal fertilizers, such as mammals, is to our knowledge unknown.

## Conclusion

Our results suggest that the combination of female age (i.e. younger females having higher reproductive success compared to older females) and MHC diversity constitutes a significant determinant of reproductive success in two groups of devils with different MHC traits and may provide an explanation for the relatively low reproductive success recorded in captive breeding programs. In order to enhance the success of this iconic species, our results advocate (i) the use of young (2 year old) female devils, and (ii) that both male and female devils are subjected to MHC genotyping with pair combinations maximizing the total numbers of heterozygous loci at opposite ends of the heterozygosity scale in order to maximize breeding success.

## Material and Methods

### Study animals

Data on pairings were obtained from the Tasmanian devil studbook^68^ and the insurance population annual reports^59–65^. Single female devils were paired with a single male when females showed signs of estrus such as a fluid filled neck roll, fat stores in the tail, reduced aggression and loss of appetite^69^. Consequently, the females were not given a choice of partner. The 37 females and 43 males used in the present study were kept across 11 institutions in Australia and the pairings were conducted from 2007 to 2012. All ear biopsies were collected by veterinarians and trained staff during the devil’s annual exams at the zoological institutions where they were housed and were hence carried out in accordance with relevant guidelines and regulations. The research was conducted under University of Sydney animal ethics approval number N00/8-2011/1/5584.

### Genetic analyses

DNA was extracted from ear biopsies using a Qiagen DNeasy® extraction kit and subsequently genotyped at six MHC Class I linked microsatellite loci described by Cheng & Belov^70^; Sh-MHCI101, Sh-MHCI102, Sh-MHCI105, Sh-MHCI106, Sh-MHCI107, and Sh-MHCI108. For further details on size range and primer sequences see Cheng & Belov^70^. Polymerase chain reactions (PCR) were carried out using the protocols of Cheng & Belov^70^ and the PCR products were quality tested on a 2% agarose gel. The samples were analyzed at the Ramaciotti Centre (University of New South Wales, Australia) and microsatellite profiles of the individual devils were subsequently scored using Peak Scanner 2.0 (Applied Biosystems 2012).

### Estimates of allele frequency, heterozygosity and linkage disequilibrium

Analyses of dropout and the presence of null alleles were conducted using the software Micro-Checker^71^. The number of alleles per locus together with observed and expected heterozygosity were estimated using the software program arlequin 3.1^72^. Tests for deviations from Hardy–Weinberg equilibrium and linkage disequilibrium, as well as the analysis of genetic structure and differentiation of the two groups were carried out using arlequin 3.1^72^ (HWE parameters included: number of steps in Markov chain = 1,000,000, number of dememorisation steps = 100,000, number of permutations = 10,000; AMOVA parameters included 999 permutations and 3000 Markov steps). Sequential Bonferroni corrections were subsequently conducted on the Hardy-Weinberg equilibrium and the linkage disequilibrium tests. Within-pair genetic similarity was conducted by recording the number of shared/identical alleles in each of the six loci.

### Statistical analyses

Logistic mixed models analyses were performed in SAS 9.4 using Proc GLIMMIX^73,74^ with institution and female ID number as random effects as 22 of the 36 females were paired on more than one occasion. Proc GLIMMIX uses restricted pseudolikelihoods and the full model included male age and identification number, number of female heterozygous loci, number of male heterozygous loci and number of similar alleles. The final model included institution as random effect and absolute number of different heterozygous loci and female age as fixed effects.
